# The Prevalence and Characteristics of Primary Headache and Dream-Enacting Behaviour in Japanese Patients with Narcolepsy or Idiopathic Hypersomnia: A Multi-Centre Cross-Sectional Study

**DOI:** 10.1371/journal.pone.0139229

**Published:** 2015-09-29

**Authors:** Keisuke Suzuki, Masayuki Miyamoto, Tomoyuki Miyamoto, Yuichi Inoue, Kentaro Matsui, Shingo Nishida, Kenichi Hayashida, Akira Usui, Yoichiro Ueki, Masaki Nakamura, Momoyo Murata, Ayaka Numao, Yuji Watanabe, Shiho Suzuki, Koichi Hirata

**Affiliations:** 1 Department of Neurology, Dokkyo Medical University, Tochigi, Japan; 2 School of Nursing, Dokkyo Medical University, Tochigi, Japan; 3 Department of Neurology, Dokkyo Medical University Koshigaya Hospital, Saitama, Japan; 4 Yoyogi Sleep Disorder Centre, Tokyo, Japan; 5 Sleep & Stress Clinic, Tokyo, Japan; 6 Kokubunji Sakura Clinic, Tochigi, Japan; Hospital General Dr. Manuel Gea González, MEXICO

## Abstract

**Background:**

Because the prevalence and characteristics of primary headache have yet to be thoroughly studied in patients with hypersomnia disorders, including narcolepsy and idiopathic hypersomnia, we examined these parameters in the Japanese population.

**Methods:**

In a multicentre cross-sectional survey, among 576 consecutive outpatients with sleep disorders, 68 narcolepsy patients and 35 idiopathic hypersomnia patients were included. Additionally, 61 healthy control subjects participated. Semi-structured headache questionnaires were administered to all participants.

**Results:**

The patients with narcolepsy (52.9%) and idiopathic hypersomnia (77.1%) more frequently experienced headache than the healthy controls (24.6%; *p*<0.0001). The prevalence rates were 23.5%, 41.2% and 4.9% for migraine (*p*<0.0001) and 16.2%, 23.5% and 14.8% (*p* = 0.58) for tension-type headache among the narcolepsy patients, the idiopathic hypersomnia patients and the control subjects, respectively. Those who experienced migraine more frequently experienced excessive daytime sleepiness, defined as an Epworth Sleepiness Scale score of ≥10, than those who did not experience headache among the patients with narcolepsy (93.8% vs. 65.6%, *p* = 0.040) and idiopathic hypersomnia (86.7% vs. 37.5%, *p* = 0.026). Dream-enacting behaviour (DEB), as evaluated by the rapid eye movement sleep disorders questionnaire, was more frequently observed in the narcolepsy patients than in the idiopathic hypersomnia patients and the control subjects. An increased DEB frequency was observed in the narcolepsy patients with migraines compared to those without headache.

**Conclusions:**

Migraines were frequently observed in patients with narcolepsy and idiopathic hypersomnia. DEB is a characteristic of narcolepsy patients. Further studies are required to assess the factors that contribute to migraines in narcolepsy and idiopathic hypersomnia patients.

## Introduction

The close relationship between sleep and headache has been recognised for over a century [1]. Headache disorders, such as migraine, tension-type headache, cluster headache and hypnic headache, are related to sleep disturbances and daytime dysfunction. Migraine attacks can be triggered by sleep deprivation or excessive sleep, and sleep relieves migraine attacks [1]. Sleep disturbance is also recognised as a risk factor for the transformation and reversion of chronic migraine [2]. In addition, an increased prevalence of headache disorders has been reported for patients with sleep disorders such as sleep apnoea syndrome and restless legs syndrome [3,4]. Based on these observations, it is possible that headache and sleep disorders share common pathophysiological mechanisms [5]. In a previous study, we found that dream-enacting behaviour (DEB) was frequently observed in migraine patients compared to healthy controls and that DEB was associated with impaired sleep and severe headache-related disability in migraine patients [6]. These observations suggest that the brainstem, which is responsible for the regulation of both rapid eye movement sleep and pain processing, may be involved in migraine.

The prevalence of narcolepsy with cataplexy has been estimated to range from 0.16% to 0.18% in Japan, and narcolepsy without cataplexy represents from 15% to 25% of the clinical narcoleptic population [7]. The prevalence of idiopathic hypersomnia is unknown. Migraine has been associated with many comorbid disorders, including depression, obesity and metabolic syndrome [8]. However, few studies have investigated comorbid primary headache and hypersomnia disorders such as narcolepsy and idiopathic hypersomnia. Dahmen et al.[9,10] observed an increased frequency of migraine (37%-54%) in patients with narcolepsy in two clinical studies; however, a multicentre study performed by another group found an increased frequency of tension-type headache (60.3% vs. 40.7%) but not migraine (21.9% vs. 19.8%) in narcolepsy patients compared with controls [11]. The frequency of primary headache has yet to be assessed in patients with idiopathic hypersomnia. Impaired quality of life has been described in patients with narcolepsy and idiopathic hypersomnia [12]. Headache comorbidity might be related to the impaired quality of life in these patients.

In this multicentre study, we aimed to evaluate the prevalence and characteristics of primary headache among Japanese patients with either narcolepsy or idiopathic hypersomnia.

## Methods

**Fig 1:**
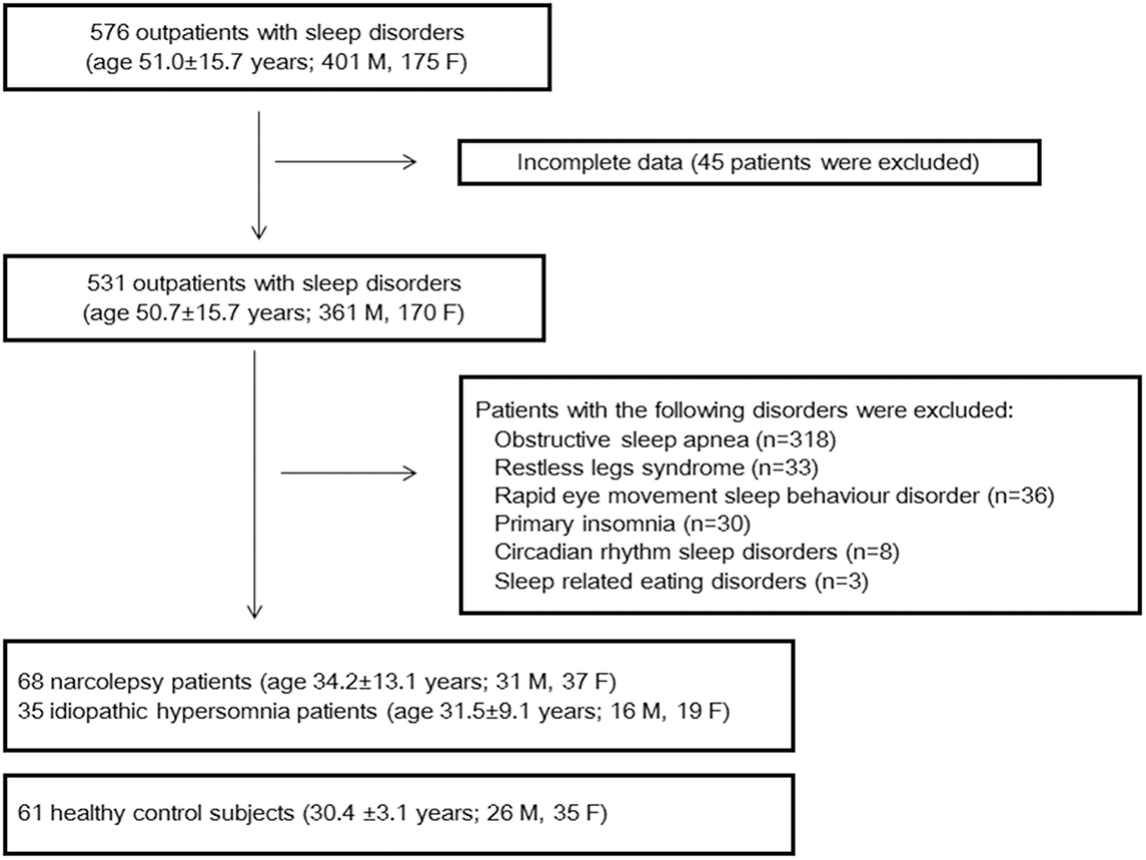
Flow chart of the patient selection method.

Fig 1 shows a flow chart of the patient enrolment process for the study. Among the 576 consecutive outpatients with sleep disorders (age, 51.0±15.7 years; 401 M, 175 F), 68 narcolepsy patients and 35 idiopathic hypersomnia patients were recruited from 5 outpatient sleep centres during the period from July 2012 to March 2013. Additionally, 61 healthy control subjects without complaints of sleep disturbances were recruited by hospital employees and friends and family.

The patients with narcolepsy or idiopathic hypersomnia were diagnosed by polysomnography and multiple sleep latency tests (MSLT) according to the International Classification of Sleep Disorders (ICSD-2), 2nd Edition. Briefly the following parameters must be met: for narcolepsy, the mean sleep latency on MSLT was <8 minutes with >2 sleep onset REM periods, and for idiopathic hypersomnia, mean sleep latency on MSLT was <8 minutes with <2 sleep onset REM periods. Patients with narcolepsy were divided into those with and without cataplexy, and patients with idiopathic hypersomnia were divided into those with (>10 hours) and without long sleep time (6–10 hours). MSLT data were available in 49 patients with narcolepsy and 34 patients with idiopathic hypersomnia. In patients with concomitant severe obstructive sleep apnea, the diagnosis of narcolepsy or idiopathic hypersomnia was determined after continuous positive airway pressure treatment.

All participants were asked to complete self-administered questionnaires regarding their habits, education and headaches. The semi-structured headache questionnaire was developed to classify chronic headache into migraine, tension-type headache, cluster headache, occipital neuralgia and other (unclassified type) according to the International Classification of Headache Disorders, 2nd Edition (ICHD-2) (S1 Table). The questionnaire included all of the essential parameters associated with migraine, tension-type headache and cluster headache, as well as a validated self-administered test for migraine headache to further improve the diagnosis of the headache disorder [13,14]. The first question was: “Have you ever had a headache?” If the answer was “yes”, the second question was: “How many times have you suffered from headaches with similar characteristics?” The following four possible answers were presented: less than 3; 3 or more; 5 or more; and 10 or more. If the patient reported experiencing 3 or more headaches with similar characteristics, they were defined as experiencing headache and were instructed to provide information about the localisation, quality, intensity, age at headache onset, headache-related disability, accompanying symptoms, presence of aura, comorbid diseases and current medications for headache.

We used the Japanese version of the Pittsburgh Sleep Quality Index (PSQI) to assess sleep disturbances, sleep quality and sleep habits [15]. The following seven component scores of the PSQI were also evaluated: C1, sleep quality; C2, sleep latency; C3, sleep duration; C4, habitual sleep efficiency; C5, sleep disturbances; C6, the use of sleeping medications; and C7, daytime dysfunction. Daytime sleepiness was evaluated using the Japanese version of the Epworth Sleepiness Scale (ESS) [16]. Excessive daytime sleepiness (EDS) was defined as a patient score of 10 or greater. Depressive symptoms were assessed using the Beck Depression Inventory-II (BDI-II) [17]. The Japanese version of the rapid eye movement sleep behaviour disorder (RBD) screening questionnaire (RBDSQ-J) is a self-rated instrument consisting of the 10 most relevant items concerning the prominent clinical characteristics of RBD [7]. An RBDSQ-J score of ≥5 indicated dream-enacting behaviour (DEB) [6,18].

This study was conducted in accordance with the Declaration of Helsinki. The study was approved by the institutional review boards of the participating centres, and all study participants provided written informed consent.

### Statistical analysis

Mann-Whitney U-tests or unpaired t-tests were used when appropriate to compare continuous variables, and chi-square or Fisher's exact tests were performed to compare the differences in frequencies between two groups. Analysis of variance and a subsequent post hoc Bonferroni test were performed to compare the differences between the narcolepsy patients, the idiopathic hypersomnia patients and the control subjects. Because our study was exploratory, we did not perform adjustments of multiplicity for multiple tests in this study [19]. Two-tailed *p* values of <0.05 were considered to be significant. IBM SPSS software version 21.0 (IBM SPSS, Inc., Tokyo, Japan) was used for statistical analyses.

## Results

Table 1 illustrates the clinical background of the patients with either narcolepsy or idiopathic hypersomnia and the control subjects. The narcolepsy and idiopathic hypersomnia patients exhibited significantly increased ESS and PSQI global scores compared to the control subjects. The narcolepsy patients exhibited increased BDI-II scores and DEB frequency (RBDSQ-J scores of ≥5) compared to the control subjects. Sleep quality was worse in the narcolepsy patients than in the idiopathic hypersomnia patients and the control subjects. The patients with idiopathic hypersomnia displayed enhanced habitual sleep efficiency compared to the patients with narcolepsy. Hypnotics were more frequently used by the narcolepsy patients than by the idiopathic hypersomnia patients and the control subjects. An increased DEB frequency was observed in the narcolepsy patients compared to the idiopathic hypersomnia patients and the control subjects. No difference in the DEB frequency was observed between the idiopathic hypersomnia patients and the control subjects.

**Table 1:** Clinical background of the narcolepsy and idiopathic hypersomnia patients and the control subjects.

	Narcolepsy	Idiopathic hypersomnia	Controls	P-value
n (M/F)	68 (31/37)	35 (16/19)	61 (26/35)	0.93
Age, years	34.2±13.1	31.4±9.2	30.4±3.1	0.080
ESS score	15.2±5.8	15.1±5.1	6.0±2.9* ^¶^	**<0.0001**
ESS≥10, n (%)	52 (76.5)	26 (74.3)	7 (11.5) * ^¶^	**<0.0001**
PSQI global score	6.8±3.1	5.2±2.8	3.6±2.2* ^¶^	**<0.0001**
PSQI component score				
C1, Sleep quality	1.5±0.8	1.2±0.8	0.8±1.0*	**<0.0001**
C2, Sleep latency	0.5±0.8	0.6±0.8	0.5±0.6	0.86
C3, Sleep duration	0.9±1.0	0.9±0.8	1.1±0.9	0.40
C4, Habitual sleep efficiency	0.3±0.7	0.03±0.2*	0.1±0.4	**0.017**
C5, Sleep disturbances	1.0±0.5	0.6±0.5	0.5±0.5*	**<0.0001**
C6, Use of sleeping medication	1.0±1.3	0.2±0.6*	0.0±0.1*	**<0.0001**
C7, Daytime dysfunction	1.9±0.9	1.8±1.0	0.5±0.6* ^¶^	**<0.0001**
BDI-II score	12.0±10.1	10.6±7.8	5.0±5.1* ^¶^	**<0.0001**
RBDSQ-J≥5, n (%)	41 (60.3)	5 (14.3) *	7 (11.5) *	**<0.0001**

Except where otherwise noted, means±SDs are given.

*p<0.05 compared to the narcolepsy patient group;

^¶^p<0.05 compared to the idiopathic hypersomnia patient group. Statistically significant values (p<0.05) are shown in bold.

ESS, Epworth Sleepiness Scale; PSQI, Pittsburgh Sleep Quality Index; BDI-II, Beck Depression Inventory-II; RBDSQ-J, the Japanese version of the rapid eye movement sleep behaviour disorder screening questionnaire.

Polysomnographic findings between patients with narcolepsy and idiopathic hypersomnia did not differ except that the arousal index was higher for narcolepsy patients (Table 2). In this study, 17.6% of narcolepsy and 25.7% of idiopathic hypersomnia patients had an apnoea-hypopnoea index (AHI) of ≥ 5.

**Table 2:** Polysomnographic findings in patients with narcolepsy and idiopathic hypersomnia.

	Narcolepsy (n = 68)	Idiopathic hypersomnia (n = 35)	p value
PSG findings			
Total sleep time (min)	486.9±64.9	473.8±60.3	0.36
Sleep latency (min)	7.7±15.8	9.0±9.0	0.67
REM latency (min)	55.2±85.1	99.8±56.5	**0.011**
WASO (min)	59.8±48.2	42.8±34.7	0.094
AHI (/h)	5.4±13.0	5.4±7.7	0.98
AHI<5, n (%)	56 (82.4)	26 (74.3)	0.34
5≤AHI<15, n (%)	9 (13.2)	5 (14.3)	
15≤AHI<30, n (%)	1 (1.5)	3 (8.6)	
AHI≥30, n (%)	2 (2.9)	1 (2.9)	
3% ODI (/h)	4.0±8.7	3.0±4.4	0.60
Arousal index (/h)	18.4±14.2	13.0±6.4	**0.048**
PLMS (/h)	5.1±14.1	1.3±3.6	0.063
MSLT findings			
Mean sleep latency≤8 min, n (%)	48/49 (98.0)	30/34 (88.2)	0.154
≥2 SOREMPs, n (%)	49/49 (100)	0/34 (0.0)	**<0.001**

Except where otherwise noted, means±SDs are provided. Statistically significant values (p<0.05) are shown in bold.

PSG, polysomnography; REM, rapid eye movement; WASO, wake after sleep onset; AHI, apnoea-hypopnoea index; ODI, oxygen desaturation index; PLMS, periodic limb movement in sleep; MSLT, multiple sleep latency test; SOREMPs, sleep-onset REM periods.

**Fig 2:**
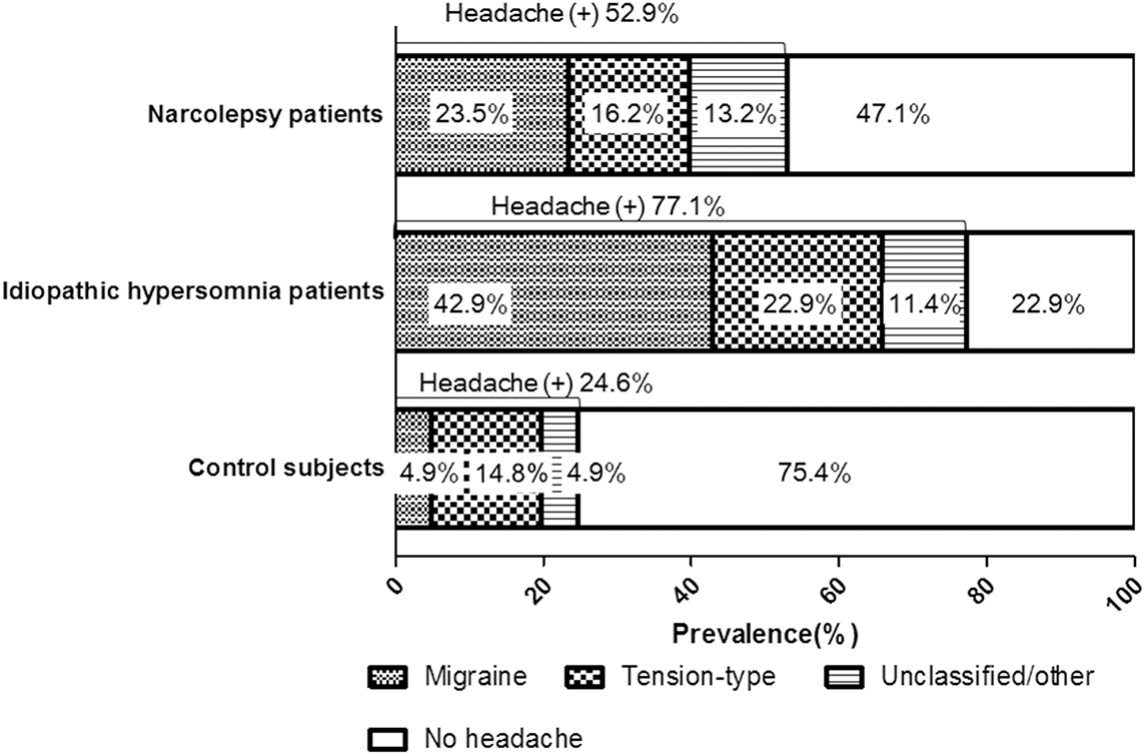
Headache prevalence in the narcolepsy patient, idiopathic hypersomnia patient and control subject groups.

Fig 2 displays the headache prevalence among the narcolepsy patients, the idiopathic hypersomnia patients and the control subjects. The patients with narcolepsy (52.9%) or idiopathic hypersomnia (77.1%) exhibited a significantly greater headache prevalence compared to the healthy controls (24.6%; *p*<0.0001). The migraine prevalence was significantly increased among the narcolepsy (23.5%) and idiopathic hypersomnia patients (41.2%) compared to the control subjects (4.9%; *p*<0.0001). The prevalence of tension-type headache tended to be increased among the idiopathic hypersomnia patients, but this difference was not significant (narcolepsy, 16.2%; idiopathic hypersomnia, 23.5%; and control subjects, 14.8%; *p* = 0.58).

An increased prevalence of EDS (ESS ≥10) was observed among the narcolepsy patients with migraine compared to those without headache (93.8% vs. 65.6%, *p* = 0.040; Table 3). The narcolepsy patients with migraine exhibited a significantly higher PSQI C7 (sleep disturbance) score than those without headache. An increased DEB frequency was observed in the narcolepsy patients with migraine compared to those without headache. Psychostimulants and anti-depressants were more frequently used in narcolepsy patients with migraine compared with those without headache. A similar tendency was observed in idiopathic hypersomnia.

**Table 3:** Characteristics of the narcolepsy patients with migraines and of those without headache.

	Narcolepsy patients with migraines (n = 16)	Narcolepsy patients without headache (n = 32)	P-value
Cataplexy, n (%)	11 (68.8)	20 (62.5)	0.67
Age, years	33±8.4	33.4±15.9	0.93
n (M/F)	16 (5/11)	32 (16/16)	0.36
ESS score	16.4±4.3	14.1±6.8	0.23
ESS≥10, n (%)	15 (93.8)	21 (65.6)	**0.040**
PSQI global score	6.7±2.7	6.4±3.3	0.76
PSQI component score			
C1, Sleep quality	1.3±0.9	1.4±0.7	0.55
C2, Sleep latency	0.7±0.7	0.5±0.8	0.37
C3, Sleep duration	0.9±1.0	1.0±0.9	0.74
C4, Habitual sleep efficiency	0.1±0.5	0.4±0.6	0.11
C5, Sleep disturbances	1.1±0.3	0.7±0.6	**0.0060**
C6, Use of sleeping medication	0.9±1.4	0.8±1.2	0.69
C7, Daytime dysfunction	1.6±1.1	1.9±0.8	0.37
BDI-II score	10.1±10.5	11.6±9.3	0.61
RBDSQ-J≥5, n (%)	14 (87.5)	13 (40.6)	**0.0023**
Psychostimulants, n (%)	10 (62.5)	7 (21.9)	**0.0055**
Antidepressants, n (%)	4 (25.0)	1 (3.1)	**0.037**

Except where otherwise noted, means±SDs are given. Statistically significant values (p<0.05) are shown in bold. ESS, Epworth Sleepiness Scale; PSQI, Pittsburgh Sleep Quality Index; BDI-II, Beck Depression Inventory-II; RBDSQ-J, the Japanese version of the rapid eye movement sleep behaviour disorder screening questionnaire.

Polysomnographic findings between narcolepsy patients with migraine and those without headache are shown in Table 4. Total sleep time was significantly shorter in narcolepsy patients with migraine compared with those without headache; however, other parameters, including the apnoea-hypopnoea index, did not differ between the groups.

**Table 4:** Polysomnographic findings in narcolepsy patients with migraine and narcolepsy patients without headache.

	Narcolepsy with migraine (n = 16)	Narcolepsy without headache (n = 32)	p-value
PSG findings			
Total sleep time (min)	457.2±50.9	500.8±54.0	**0.022**
Sleep latency (min)	12.7±25.8	6.4±13.8	0.32
REM latency (min)	41.7±43.7	48.3±75.2	0.78
WASO (min)	63.0±37.0	50.7±44.6	0.41
AHI (/h)	3.2±3.2	7.0±16.9	0.45
AHI<5, n (%)	14 (87.5)	24 (75.0)	0.69
5≤AHI<15, n (%)	2 (12.5)	6 (18.8)	
15≤AHI<30, n (%)	0 (0.0)	1 (3.1)	
AHI≥30, n (%)	0 (0.0)	1 (3.1)	
3% ODI (/h)	2.4±3.1	5.0±11.4	0.45
Arousal index (/h)	18.7±9.0	18.9±15.7	0.96
PLMS (/h)	7.8±21.3	5.7±14.1	0.71
MSLT findings			
Mean sleep latency≤8 min, n (%)	9/9 (100)	26/27 (96.3)	1.0
≥2 SOREMPs, n (%)	9/9 (100)	27/27 (100)	

Except where otherwise noted, means±SDs are provided. Statistically significant values (p<0.05) are shown in bold.

PSG, polysomnography; REM, rapid eye movement; WASO, wake after sleep onset; AHI, apnoea-hypopnoea index; ODI, oxygen desaturation index; PLMS, periodic limb movement in sleep; MSLT, multiple sleep latency test; SOREMP, sleep-onset REM periods.

No significant difference in the headache prevalence was observed between the narcolepsy patients with cataplexy (n = 44) and those without cataplexy (n = 24; headache, 45.5% vs. 50.0%: migraine, 25.0% vs. 20.8%; tension-type headache, 18.2% vs. 12.5%). The patients with idiopathic hypersomnia displayed no difference in clinical background or in the scores on the questionnaires, except that EDS was more prevalent among the idiopathic hypersomnia patients with migraine compared to those without headache (86.7% vs. 37.5%, *p* = 0.026; Table 5). The headache prevalence did not significantly differ between the idiopathic hypersomnia patients with long sleep time (n = 9) and those without long sleep time (n = 26; headache, 62.5% vs. 81.5%; migraine, 25.0% vs. 48.1%; tension-type headache, 25.0% vs. 22.2%).

**Table 5:** Characteristics of the idiopathic hypersomnia patients with migraines and those without headache.

	Idiopathic hypersomnia patients with migraines (n = 15)	Idiopathic hypersomnia patients without headache (n = 8)	P-value
Long sleep time, n (%)	2 (13.3)	3 (37.5)	0.30
Age, years	30.3±8.7	31.9±9.3	0.69
n (M/F)	15 (4/11)	8 (4/4)	0.37
ESS score	16.6±4.4	12.1±7.2	0.084
ESS≥10, n (%)	13 (86.7)	3 (37.5)	**0.026**
PSQI global score	5.5±2.8	4.3±2.3	0.27
PSQI component score			
C1, Sleep quality	1.3±0.9	1.0±0.8	0.48
C2, Sleep latency	0.4±0.7	0.8±0.9	0.32
C3, Sleep duration	0.9±0.7	0.5±0.5	0.23
C4, Habitual sleep efficiency	0.1±0.3	0.01±0.02	0.48
C5, Sleep disturbances	0.6±0.5	0.6±0.5	0.91
C6, Use of sleeping medication	0.5±1.1	0.1±0.4	0.39
C7, Daytime dysfunction	1.9±1.0	1.3±1.0	0.18
BDI-II score	11.1±10	9.1±7.8	0.64
RBDSQ-J≥5, n (%)	3 (20.0)	0 (0.0)	0.53
Psychostimulants, n (%)	8 (53.3)	2 (25.0)	0.38
Antidepressants, n (%)	3 (20.0)	0 (0.0)	0.52

Except where otherwise noted, means±SDs are given. Statistically significant values (p<0.05) are shown in bold. ESS, Epworth Sleepiness Scale; PSQI, Pittsburgh Sleep Quality Index; BDI-II, Beck Depression Inventory-II; RBDSQ-J, the Japanese version of the rapid eye movement sleep behaviour disorder screening questionnaire.

Polysomnographic findings in idiopathic hypersomnia with migraine and those without headaches were not significantly different (Table 6).

**Table 6:** Polysomnographic findings in idiopathic hypersomnia patients with migraine and idiopathic hypersomnia patients without headache.

	Idiopathic hypersomnia with migraine (n = 15)	Idiopathic hypersomnia without headache (n = 8)	p-value
PSG findings			
Total sleep time (min)	489.9±50.2	457.8±63.9	0.24
Sleep latency (min)	6.7±3.1	12.6±13.5	0.27
REM latency (min)	92.1±34.8	120.6±92.5	0.43
WASO (min)	40±41.6	50.3±34	0.57
AHI (/h)	2.8±4.9	5.5±6.2	0.31
AHI<5, n (%)	14 (93.3)	5 (62.5)	0.12
5≤AHI<15, n (%)	0 (0.0)	2 (25.0)	
15≤AHI<30, n (%)	1 (7.1)	1 (12.5)	
AHI≥30, n (%)	0 (0.0)	0 (0.0)	
3% ODI (/h)	1.3±1.9	4.8±5.8	0.21
Arousal index (/h)	10.8±4.3	14.3±8.5	0.26
PLMS (/h)	1.8±5.3	0.6±1.6	0.58
MSLT findings			
Mean sleep latency≤8 min, n (%)	13/14 (92.9)	7/8 (87.5)	1.0
≥2 SOREMPs, n (%)	0/14 (0.0)	0/8 (0.0)	

Except where otherwise noted, means±SDs are provided.

PSG, polysomnography; REM, rapid eye movement; WASO, wake after sleep onset; AHI, apnoea-hypopnoea index; ODI, oxygen desaturation index; PLMS, periodic limb movement in sleep; MSLT, multiple sleep latency test; SOREMP, sleep-onset REM periods.

Psychostimulants were used by 27 (39.7%) narcolepsy patients and by 14 (40.0%) idiopathic hypersomnia patients. Antidepressants were used by 10 (14.7%) patients with narcolepsy and by 3 (8.6%) patients with idiopathic hypersomnia. Among all of the migraine patients, 37 patients and 2 patients were treated with nonsteroidal anti-inflammatory drugs and triptans, respectively, when headache occurred. However, among the patients with tension-type headache, 9 were treated with nonsteroidal anti-inflammatory drugs.

## Discussion

This is the first multicentre study in Japan examining a potential relationship between hypersomnia disorders and headache that has demonstrated an increased headache prevalence among patients with narcolepsy or idiopathic hypersomnia compared to healthy controls (52.9%, 77.1% and 24.6%, respectively; Fig 2). Additionally, the migraine prevalence was higher in the patients with narcolepsy and idiopathic hypersomnia compared to healthy controls (23.5%, 42.9% and 4.9%, respectively; Fig 2). The frequency of cataplexy among the narcolepsy patients observed in this study (68.8%) was lower than that found in previous studies (Dahmen et al., 95–100% [9,10]; DMKG study, 80% [11]). However, the tension-type headache prevalence was not significantly different between the groups. The increased prevalence of migraine among the narcolepsy and idiopathic hypersomnia patients in our study was clear when considering the results of a nationwide survey that included 4029 subjects aged 15 years or older in Japan, which reported an overall migraine prevalence of 8.4% [20].

Similarly, Dahmen et al.[9,10] reported a significantly increased prevalence of migraine (37–44%) among narcolepsy patients, although that study did not include a control group. In their study, migraine occurred after the onset of narcolepsy in most patients [9]. The authors attributed the increased migraine prevalence to a common pathophysiology relevant to both disorders that included immunological processes and the brainstem, such as serotonergic systems, rather than simply to the sleep disturbances observed in narcolepsy patients. In contrast to our observation, another study showed a similar migraine frequency between narcolepsy patients (21.9%) and control subjects (19.8%), although tension-type headache was more prevalent among the narcolepsy patients than the control subjects (60.3% vs. 40.7%) [11]. The authors proposed that patients with narcolepsy may experience an increased frequency of unspecified types of headaches other than migraines that result from sleep disturbances.

The dysregulation of REM sleep is closely related to the clinical characteristics of narcolepsy, including sleep-onset REM periods, cataplexy, sleep paralysis and hypnagogic hallucinations that likely result from the intrusion of REM sleep into wakefulness, but is not closely related to the characteristics of idiopathic hypersomnia [21]. Similarly, it has been suggested that REM sleep is involved in migraine attacks based on the observations that recurrent vivid dreams are associated with migraine attacks [22], migraine attacks frequently occur during REM sleep [23], and migraine patients exhibit hallucinations [24,25], increased REM sleep and prolonged REM sleep latencies [26]. In our study, the results that an increased frequency of RBDSQ-J scores ≥5 is observed in the narcolepsy group are in accordance with the finding that DEB and RBD occur more frequently in patients with narcolepsy [27].

In our study, comorbid migraine was associated with EDS both in patients with narcolepsy and in patients with idiopathic hypersomnia. In addition, the sleep disturbance score (PSQI component score 5) was higher in narcoleptic patients with migraine than in those without headache; however, this score was similar for idiopathic hypersomnia patients with migraine and for those without headache. Our findings may indicate that migraine increased RBD-related symptoms and interfered with sleep, resulting in additional daytime sleepiness among narcolepsy patients. Poor sleep quality, difficulty initiating and maintaining sleep and EDS were observed more frequently among migraine patients compared with healthy controls [4,28,29] Migraine attacks vary with seasonal, menstrual and circadian rhythms, which suggests that chronobiological rhythms may be impaired in patients with migraine [30]. Therefore, it is also possible that poor sleep quality related to comorbid migraine may worsen the fragmentation of night-time sleep commonly seen in narcolepsy patients but not in idiopathic hypersomnia patients. By contrast, among idiopathic hypersomnia patients, the lack of association between EDS and sleep status suggests that migraine may directly affect the maintenance of wakefulness in idiopathic hypersomnia. Because AHI did not differ between narcolepsy patients with migraine and those without headache or between idiopathic hypersomnia patients with migraine and those without headache, we suggest that sleep apnoea did not contribute to increased sleepiness in narcolepsy and idiopathic hypersomnia patients with comorbid migraine.

Decreased levels of orexins, which are wake-promoting neuropeptides, have been detected in the cerebrospinal fluid (CSF) of 95% of narcoleptic patients with cataplexy and of 41% of patients with narcolepsy without cataplexy [21]. Several studies have demonstrated the anti-nociceptive properties of orexins [31], although a pro-nociceptive effect of orexins has also been reported [32]. A significant association between migraine headaches without aura and the orexin (hypocretin) receptor 1 gene has been reported [33]. By contrast, higher levels of orexin-A were found in the CSF of chronic migraine and medication-overuse headache patients compared to the CSF of control subjects, suggesting that a compensatory response to chronic pain or a hypothalamic response to stress may occur in these patients [34]. A recent experimental study showed that a dual antagonist of both orexin receptors 1 and 2 attenuated neurogenic dural vasodilation and trigeminocervical complex activation and reduced susceptibility to KCl-evoked cortical spreading depression in animal models of migraine [35].

Previous studies have described normal CSF levels of orexin [36] in idiopathic hypersomnia patients and reduced CSF levels of histamine in orexin-deficient narcolepsy and idiopathic hypersomnia patients [37]. Histamine plays an important role in the sleep-wake cycle by consolidating the awake state and by promoting cortical excitability during the awake state. Centrally acting H3 receptor agonists cause the autoinhibition of histaminergic neurons, which inhibits neurogenic inflammation in the dura, induces sleep and produces anti-nociception [38]. This observation suggests that the histaminergic system of the brain may play an important role in migraine attacks.

One limitation of this study was that although the assessments included a migraine screening questionnaire that was based on the ICHD-2 criteria, we relied on headache questionnaires for the diagnosis of migraine and tension-type headache. Because of the questionnaire-based nature of this study, we could not evaluate the overlap of migraine and tension-type headaches in this study. Second, polysomnography was not performed in control subjects, although they had no sleep complaints. Third, we could not assess the relationship between the onset of migraines and the onset of idiopathic hypersomnia or narcolepsy, as most patients could not recall the exact moment of onset. Forth, the HLA-DR2 status, which has been implicated in migraine, was not assessed in this study. Narcolepsy with and without cataplexy may involve different pathophysiologies based on differences in the CSF orexin levels and in the HLA DQB1*0602 status between these disorders. Therefore, ICSD-3 narcolepsy was categorised into narcolepsy type 1 and type 2 and was predicated on the concept that an absence of orexin is a fundamental marker of narcolepsy. Idiopathic hypersomnia with and without long sleep time may also involve different pathophysiologies [39]. Nevertheless, in our study, no difference in the prevalence of migraine was found between narcolepsy patients with cataplexy and those without cataplexy or between idiopathic hypersomnia patients with long sleep time and those without long sleep time. In previous studies, sleep loss and oversleeping have both been associated with headache;[1,40] however, long sleep time was not related to migraine occurrence among patients with idiopathic hypersomnia in our study. Finally, psychostimulants and anti-depressants were more frequently used in narcolepsy and idiopathic hypersomnia patients with migraines compared with those without headaches. Therefore, although drug effects on sleepiness in the migraine group are difficult to interpret, we believe that migraine comorbidity itself may have had an impact on sleepiness in this study.

In conclusion, migraines were frequently observed in narcolepsy and idiopathic hypersomnia patients. Further studies that recruit a large number of narcolepsy and idiopathic hypersomnia patients are required to evaluate the factors that contribute to migraines in hypersomnia disorders.

## Supporting Information

S1 Table Headache questionnaire. (DOC)
