# Supplementary material for: The Prevalence and Characteristics of Primary Headache and Dream-Enacting Behaviour in Japanese Patients with Narcolepsy or Idiopathic Hypersomnia: A Multi-Centre Cross-Sectional Study
Source: PLoS One. 2015 Sep 29;10(9):e0139229. doi: 10.1371/journal.pone.0139229 (PMC4587931; doi:10.1371/journal.pone.0139229)
Supplement: S1 Table — (DOC) [file pone.0139229.s001.doc]

**Supporting Information**

**Supplementary Table 1**

**Headache questionnaire**

**Date： 　　　Signature**

**Age**：　 　　(y), **Birth date**：

**Gender**：　□ Male　□ Female

- **Have you ever had a headache?**

□　Yes→ please continue with Q1

□　No→ please go to the end of the questionnaire

**Q1**　 How many times have you suffered from headaches with similar characteristics?

□　Less than 3 → please go to the end of the questionnaire

□　3 or more → please continue with Q2

□　5 or more → please continue with Q2

□　10 or more → please continue with Q2

**Q2**　At what age did your headaches start?　 Age [　　　] years old

**Q3**　Please answer the following four questions regarding your headaches over the previous three months:

|  | Pain is made worse by activities such as walking or climbing stairs? □Never　□Rarely　□Less than half the time　□Half the time or more |
| --- | --- |
|  | You feel nauseated or sick to your stomach □Never　□Rarely　□Less than half the time　□Half the time or more |
|  | Light bothers you (a lot more than when you don’t have headaches) □Never　□Rarely　□Less than half the time　□Half the time or more |
|  | Smell bothers you (a lot more than when you don’t have headaches) □Never　□Rarely　□Less than half the time　□Half the time or more |

**Q4**　 When did the headaches tend to occur?

□　Morning

□　Noon

□　Evening

□　During sleep

□　Not specified (any time)

**Q5**　How long did the headaches last?

□　A few seconds to a few minutes

□　A few minutes to 30 minutes

□　30 minutes to 3 hours

□　4 to 72 hours (3 days)

□　1 week or more

**Q6** In which part of the head did you have the headache?

□　Deep behind the eye (s)

□　Around the eye (s)

□　Back of the head to neck

□　Front part

**Q7**　 Did your headache occur on one side of the head or both sides?

□　One side

□　Both sides

**Q8**　What was your headache quality?

□　Pulsating or throbbing

□　Pressing or tightening

□　Stabbing

□　Lightning or electric shock sensations

**Q9**　How severe was your headache?

□　Intolerable, difficult to perform usual daily activities (very severe pain)

□　Headaches limit your ability to work or study (moderate to severe pain)

□　Tolerable (mild to moderate pain)

**Q10**　Did you have the following symptoms when the headaches occurred?

□　Pain was made worse by daily activities

□　Needed to go to bed

□　Headaches relieved with movement

□　Nausea or vomiting

□　Light bothered you or felt discomfort from lights

□　Sound bothered you

□　Smell bothered you

□　A sense of restlessness or agitation during headache

**Q11**　Did you have the following symptoms before the onset of headache?

|  | Yes | No |
| --- | --- | --- |
| - Visual aura   Seeing flashing lights, lines or dots or having visual field defects | □ | □ |
| - Sensory aura   Tingling or numbness on one side of the head or body | □ | □ |
| - Hemiplegia   Unable to move the hand and/or leg on one side. | □ | □ |
| - Aphasia   Unable to speak and understand speech | □ | □ |
| - How long did the following symptoms last? | (　　　　) minutes | |
| - - Have you suffered from this type of headache previously five times or more?   Yes □  No　□　→　 (　　　　) times | | |

**Q12**　Did you have the following accompanying symptoms on the headache side?

- Conjunctival injection and/or lacrimation 　□ Yes　　□ No
- Nasal congestion and/or rhinorrhea 　 □ Yes　　□ No
- Ptosis or eyelid edema 　□ Yes　　□ No
- Sweating on the forehead or face 　□ Yes　　□ No

**Q13**　Were the headaches related to your menstrual cycle (for women only)?

□ Yes　　□ No

Please describe anything you want to tell us.

( )

**Medication**

Please list all current drugs that you take for your headaches.

| **Drug name** | Days per month　(over the past three months) | Daily dose  (over the past three months) | Treatment duration |
| --- | --- | --- | --- |
| 1 | （　　　　）days/month | （　　　　　　）/day | （　　　　）  years |
| 2 | （　　　　）days/month | （　　　　　　）/day | （　　　　）  years |
| 3 | （　　　　）days/month | （　　　　　　）/day | （　　　　）  years |

Please list all current drugs that you take daily to prevent your headaches.

| **Drug name** | Daily dose  (over the past three months) | Treatment duration |
| --- | --- | --- |
| 1 | （　　　　　　）/day | （　　　　）years |
| 2 | （　　　　　　）/day | （　　　　）years |
| 3 | （　　　　　　）/day | （　　　　）years |

Please list all current drugs that you take for other diseases

| **Drug name** | Daily dose  (over the past three months) | Treatment duration |
| --- | --- | --- |
| 1 | （　　　　　　）/day | （　　　　）years |
| 2 | （　　　　　　）/day | （　　　　）years |
| 3 | （　　　　　　）/day | （　　　　）years |
